# Recruitment and retention in internet based randomised trials

**DOI:** 10.1186/1745-6215-14-S1-O113

**Published:** 2013-11-29

**Authors:** Gillian W Shorter, Finola Ferry

**Affiliations:** 1 MRC All Ireland Hub for Trials Methodology Research, University of Ulster, Londonderry, UK; 2 Bamford Centre for Mental Health and Wellbeing, University of Ulster, Londonderry, UK

## Introduction

The use of the internet to recruit participants, deliver interventions or collect data in randomised controlled trials (RCTs) is increasing and offers many potential advantages. However, there are limitations in identifying and recruiting participants, and higher loss to follow up than conventional trials.

## Methods

A systematic review of internet based RCTs was conducted including those conducted either fully or primarily on the internet in the area of alcohol brief interventions. The review (on-going) aimed to identify the challenges, compare the rates and methods of recruitment and retention, and note any methods particularly successful in meeting the challenges of recruitment and retention in online RCTs.

## Results

Commonly recruitment was through non-personal communication such as search engines, media or websites, but there was a role for personal interaction such as in person or email recruitment. Particular advantages were reaching non-clinical or hard-to-reach populations. However, online recruitment can be unrepresentative, with multiple or unverified identities common, and those recruited may not fully participate in the intervention. Regarding retention, few studies had a high follow up rate, with rates as low as 10% in some studies. This was often explained by time constraints, changes in circumstances or problems with the study itself. Few solutions were offered, but offline consent and data collection was proposed to improve retention rates.

## Conclusion

Internet based RCTs are advantageous in reducing costs and improving equity of access to research. Strategies to improve include better support and connection with participants, and increased consultation with participants about their views.

